# Comparative Composition and Antioxidant Activity of Peptide Fractions Obtained by Ultrafiltration of Egg Yolk Protein Enzymatic Hydrolysates

**DOI:** 10.3390/membranes1030149

**Published:** 2011-07-06

**Authors:** Bertrand P. Chay Pak Ting, Yoshinori Mine, Lekh R. Juneja, Tsutomu Okubo, Sylvie F. Gauthier, Yves Pouliot

**Affiliations:** 1 Institute of Nutraceuticals and Functional Foods (INAF), Department of Food Science and Nutrition, Université Laval, Québec, QC, G1V 0A6, Canada; E-Mails: bertrand.chay-pak-ting.1@ulaval.ca (B.P.C.P.T.); sylvie.gauthier@fsaa.ulaval.ca (S.F.G.); 2 Department of Food Science, University of Guelph, Guelph, ON, N1G 2W1, Canada; E-Mail: ymine@uoguelph.ca; 3 Research Laboratories, Taiyo Kagaku Co, Ltd., 1-3 Takaramachi, Yokkaichi, Mie 510 0844, Japan; E-Mails: ljuneja@taiyokagaku.co.jp (L.R.J.); tokubo@taiyokagaku.co.jp (T.O.)

**Keywords:** egg yolk proteins, enzymatic hydrolysis, ultrafiltration, antioxidant activity

## Abstract

The objective of the study was to compare the antioxidant activity of two distinct hydrolysates and their peptide fractions prepared by ultrafiltration (UF) using membranes with molecular weight cut-off of 5 and 1 kDa. The hydrolysates were a delipidated egg yolk protein concentrate (EYP) intensively hydrolyzed with a combination of two bacterial proteases, and a phosphoproteins (PPP) extract partially hydrolyzed with trypsin. Antioxidant activity, as determined by the oxygen radical absorbance capacity (ORAC) assay, was low for EYP and PPP hydrolysates with values of 613.1 and 489.2 μM TE·g^−1^ protein, respectively. UF-fractionation of EYP hydrolysate increased slightly the antioxidant activity in permeate fractions (720.5–867.8 μM TE·g^−1^ protein). However, ORAC values were increased by more than 3-fold in UF-fractions prepared from PPP hydrolysate, which were enriched in peptides with molecular weight lower than 5 kDa. These UF-fractions were characterized by their lower N/P atomic ratio and higher phosphorus content compared to the same UF-fractions obtained from EYP-TH. They also contained high amounts of His, Met, Leu, and Phe, which are recognized as antioxidant amino acids, but also high content in Lys and Arg which both represent target amino acids of trypsin used for the hydrolysis of PPP.

## 1. Introduction

Antioxidants have been traditionally used in the food processing industry to prevent lipid oxidation which causes deteriorations of food quality, confers unacceptable taste and shortens shelf-life [1,2]. However, over the last decades, it has been evidenced that antioxidants could also have physiological effects in humans by preventing damage to cellular components arising as a consequence of chemical reactions involving free radicals [3,4].

A large number of food protein-derived peptides have been shown to possess antioxidant properties [5]. Some naturally-occurring peptides exhibit strong antioxidant activity due to their hydrophobic character and their ability to interact with lipids [6]. Such antioxidant properties have been related to hydrophobic amino acid residues Val or Leu at the N-terminus of these peptides, but also to the presence of amino acids such as Pro, His or Tyr in their sequences [7,8,9]. In fact, several amino acids have been considered as antioxidants in spite of their pro-oxidative effects in some cases [10]. Although the structure-activity relationship of antioxidative peptides, such as His-containing species, has not been well defined yet, the activity could be attributed to hydrogen-donor ability, lipid peroxy-radical trapping and/or the strong metal chelating activity owing to the decomposition of the imidazole group [11]. Such antioxidant amino acids can donate proton or hydrogen to reactive oxygen species and thus generate radical quenching activities [12].

Egg yolk proteins (EYP) have been found to possess antioxidant activity in a linoleate emulsion system [13] and phosvitin, a highly phosphorylated protein among EYP, has been shown to possess a very strong metal chelating capacity [14,15]. Also, it has been suggested that EYP hydrolysates could be used as free-radical scavenging agents in a linoleic acid oxidation system [16,17,18]. The antioxidant activities of egg proteins and of their hydrolysates thus offer the potential application to enhance stability against oxidative deterioration while having the additional advantages of improving their nutritional and potential health values [17,18,19].

Delipidated EYP is a by-product of lecithin extraction from egg yolk in the egg-processing industry. However, the delipidation process using ethanol and hexane for removal of phospholipids impairs protein functionality and delipidated EYP display reduced solubility properties [20]. Enzymatic hydrolysis thus represents a valuable approach to process protein-rich egg yolk streams into products having biological properties.

Phosphopeptides having molecular masses between 1 and 3 kDa, obtained by tryptic hydrolysis of phosvitin, have been found to be effective for enhancing calcium binding capacity and to inhibit the formation of insoluble calcium phosphate [21,22]. These phosphopeptides have also been shown to have free radical scavenging and antioxidant activities against lipid peroxidation and were also showed to be effective *in vitro* against oxidative stress in an assay using human intestinal epithelial cells [23]. Jiang and Mine [24] have developed laboratory-scale experimental conditions to separate phosphoproteins (PPP) from delipidated EYP. Chay Pak Ting *et al.* [25] developed and scaled-up a membrane-based approach using ultrafiltration (UF) for the production of PPP from a commercial delipidated EYP.

More recently Young *et al.* [26] investigated a number of enzymes and combination of bacterial proteases in order to produce EY phosphopeptides having antioxidative properties. Crude EY phosphopeptides, obtained by enzymatic hydrolysis using Alcalase and Protease N (both from *Bacillus subtilis*), showed antioxidant activity, supporting the view that delipidated EYP could be considered as a source of novel antioxidative peptides. These findings also suggest that biological activities of protein hydrolysates can be related to synergistic contributions of amino acid composition, sequence and molecular weight of peptides.

UF-membrane-based separations of enzymatic hydrolysates can achieve the removal of peptides from non-hydrolyzed proteins and proteolytic enzymes [27]. In addition, UF can also be used to perform peptide separation according to their molecular mass and also to their charge. For example, it has been shown that UF-separation of casein peptide mixtures could generate permeates having different amino acid composition due to rejective properties of the membrane towards charged or hydroxylated amino acid residues [28,29].

The objective of this study was to evaluate the impact of UF-fractionation on the antioxidant activity of two different enzymatic hydrolysates, the first one prepared from EYP and extensively hydrolyzed with a combination of two bacterial proteases, and the second one produced from PPP and partially hydrolyzed using trypsin. Both hydrolysates were further fractionated by a two-step UF-process using membranes with low molecular weight cut-off (5 and 1 kDa) to obtain UF-fractions with distinctive composition. The antioxidant activity of the hydrolysates and their UF-fractions were then assessed using the oxygen radical absorbance capacity (ORAC) assay.

## 2. Experimental Section

### 2.1. Materials and Chemicals

Defatted egg yolk protein concentrate was a gift from Taiyo Kagaku Co. Ltd. (Yokkaichi, Japan). Alcalase (from *Bacillus subtilis*, EC 3.4.21.62, HBI Enzymes Inc., Osaka, Japan) and protease N (from *B. Subtilis*, 150 ATEE U·mg^−1^, EC 3.4.24.28, Amano Enzymes, Elgin, IL, USA) were kindly provided by Dr Mine's laboratory (University of Guelph, Guelph, ON, Canada). Trypsin VI from bovine pancreas, containing 2800 U·mg^−1^ of trypsin activity (EC 3.4.21.4) and 490 U·mg^−1^ of chymotrypsin activity (EC 3.4.21.4), was donated by Neova Technologie Inc. (Abbotsford, BC, Canada). All other chemicals used in the experiments were of analytical grade.

### 2.2. Preparation of Phosphoproteins (PPP) from Egg Yolk Protein (EYP)

Preparation of PPP from EYP was performed as described in Chay Pak Ting *et al.* [25]. Briefly, EYP was suspended in 10% NaCl and centrifuged to remove the insoluble materials. The supernatant was ultrafiltered using a pilot scale module Lab Unit 1812 (Filtration Engineering Co., Inc., Champlin, MN, USA) with a 30 kDa molecular weight cut-off (MWCO) membrane having a filtering area of 0.32 m^2^. The solution was concentrated until a volume concentration ratio (VCR) of 6X and followed by a diafiltration (DF) step using 10 diavolumes (DV) of water. The final retentate, so-called PPP, was lyophilized and stored at −35 °C until use.

### 2.3. Preparation of Enzymatic Hydrolysates and Their UF-Fractions

**Figure 1:**
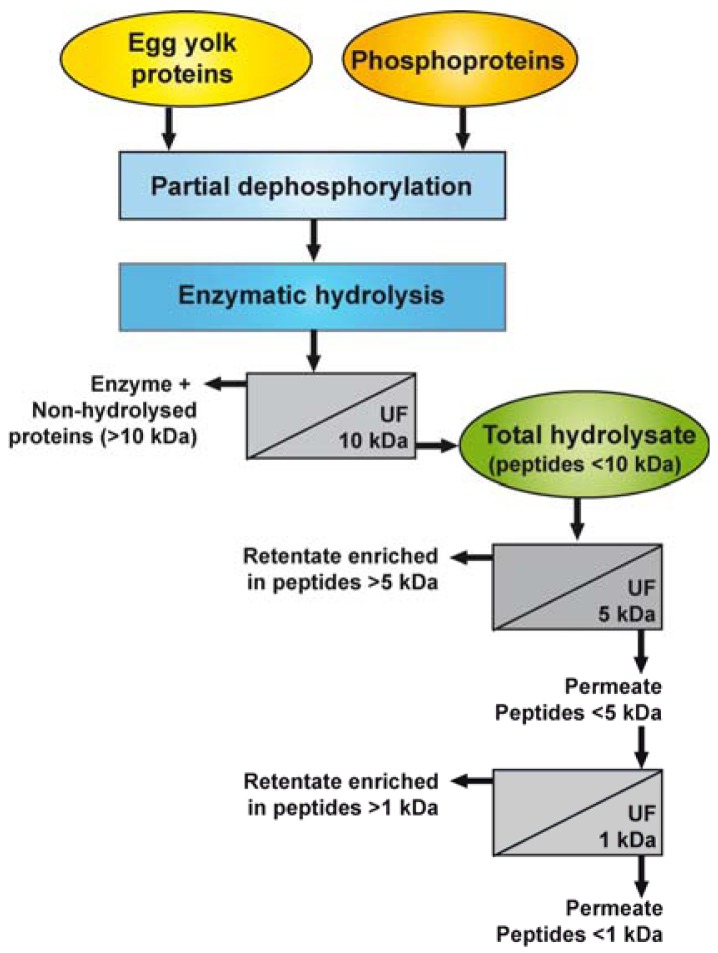
Schematic representation of the process used for the dephosphorylation of EYP and PPP, the production of enzymatic hydrolysates and their UF-fractions.

Figure 1 illustrates the different steps used for the dephosphorylation of EYP and PPP and the preparation of the enzymatic hydrolysates and their UF-fractions. Prior to the hydrolysis reaction, EYP and PPP were rehydrated in 0.1 N NaOH (5%, w/v) and partially dephosphorylated by incubating the solution at 37 °C for 3 h, as previously described [25].

Enzymatic hydrolysis of dephosphorylated EYP solution was performed as described by Young *et al.* [26]. Briefly, the EYP solution (pH 13.0) was adjusted to pH 10 with 1 N HCl, then Alcalase (0.5%, w/w) was added and the solution was maintained at 45 °C for 3 h under constant stirring. Thereafter, the reaction mixture (pH 7.6) was re-adjusted to pH 8.0 using 2 M NaOH, Protease N (0.5%, w/w) was added then the solution was held at 45 °C under constant stirring for 16 h.

Dephosphorylated PPP solution was hydrolyzed with trypsin as described by Jiang and Mine [22]. The solution was first adjusted to pH 8.0 with 1 N HCl then trypsin VI was added at a E:S ratio of 1:50. During the hydrolysis, the reaction mixture was held at 45 °C and maintained at pH 8.0 by adding 2 N NaOH to determine the degree of hydrolysis (DH). The reaction was considered complete when stable DH values were obtained.

Both enzymatic hydrolysis reactions (EYP and PPP) were stopped by UF-separation using a polyethersulfone 10 kDa MWCO membrane to remove the enzymes and non-hydrolysed proteins. The permeates, so-called total hydrolysates (EYP-TH and PPP-TH), were lyophilized.

EYP-TH and PPP-TH were rehydrated in water (1%, w/w) then fractionated consecutively through UF membranes having MWCO of 5 and 1 kDa (Figure 1). The first UF separation was performed using a regenerated cellulose 5 kDa-membrane (Prep/scale™-TFF, 0.11 m^2^, Millipore Corp., Bedford, MA, USA) at a transmembrane pressure of 1.38 bar until a VCR of 5X was reached. The permeate collected in this first stage was further ultrafiltered using a regenerated cellulose 1 kDa-membrane coupon with an effective area of 0.09 m^2^, which was mounted on a SEPA ST system (GE Osmonics, Minnetonka, MN). UF was performed at a transmembrane pressure of 2.76 bars until a VCR of 4X was reached. This process yielded four fractions for each TH: a retentate enriched in peptides >5 kDa (UF5-R), a permeate mainly composed of peptides <5 kDa (UF5-P), a second retentate enriched in peptides >1 kDa (UF1-R), and a second permeate containing peptides <1 kDa. All retentates and permeates were freeze-dried and stored at −35 °C until further analysis.

### 2.4. Physicochemical Characterization of the Enzymatic Hydrolysates and Their UF-Fractions

The degree of hydrolysis (DH, %) of EYP hydrolysate was determined in final powder by the ratio AN/TN, where AN is the amino-nitrogen content as determined using the formaldehyde titration method [30], and TN is the total nitrogen content which was determined by the Dumas combustion method described below. For tryptic hydrolysis of PPP, DH was measured during the enzymatic reaction by the pH-Stat technique [31].

The solubility of EYP-TH and PPP-TH was measured by the nitrogen solubility index (NSI) method [32] over the pH range from 2 to 10. The desired pH of sample solution was adjusted using 1 N NaOH or HCl. The solution was agitated for 2 h at 30 °C then centrifuged at 3,000 × *g* for 10 min. The supernatant was decanted and passed through a Whatman No. 1 filter paper. Total nitrogen content was determined in filtrate and initial solution as described below. NSI value is expressed in percentage and represents the nitrogen content in filtrate divided by the nitrogen content in initial solution.

Molecular weight distribution of EYP-TH, PPP-TH and their UF-fractions were determined by gel permeation chromatography [29] using a high-performance liquid chromatography system (Agilent Technologies system, 1100 series, Palo Alto, CA, USA) with a TSK gel column G2000SW_XL_ (7.8 mm × 300 mm, Tosoh). A molecular weight calibration curve was prepared from the average retention time of protein/peptide standards ranging from 654 Da to 78 kDa.

The nitrogen content in samples was determined by the Dumas combustion method using a LECO FP-528 apparatus (model 601-500, LECO Corporation, St Joseph, MI, USA). Protein content was calculated from the total nitrogen content of samples using a protein conversion factor of 6.25.

Phosphorus content was determined by the colorimetric molybdenum method of Allen [33] after acid digestion of the samples using 6 N perchloric acid.

Amino acid composition was determined as Diaz *et al.* [34] after acid hydrolysis of the samples with HCl 6 N for 24 h at 110 °C.

### 2.5. Measurement of Oxygen Radical Absorbance Capacity (ORAC)

The ORAC assay was performed as described by Cao *et al.* [35]. The dried samples of EYP-TH, PPP-TH and their UF-fractions were dissolved in 0.075 M phosphate buffer (pH 7.0) at 37 °C. Trolox, a water-soluble analogue of vitamin E, was used as control standard. For the analysis, fluorescein was used as a target of free radical attack with 2,2-azobis(2-amidinopropane) dihydrochloride (AAPH) as a source for the peroxyl radical, which is generated as a result of the spontaneous decomposition of AAPH at 37 °C. The automated ORAC assay was carried out with a Galaxy fluorometer (BGM LabTech, Durham, NC, USA). The sample's fluorescence was recorded 35 times during 120 min at emission and excitation wavelengths of 520 and 485 nm, respectively. The ORAC values are expressed as μmol of Trolox equivalents (TE) per gram of protein.

### 2.5. Statistical Analysis

Statistical differences between antioxidant activity of enzymatic hydrolysates and their respective UF-fractions were determined by one-way analysis of variance (ANOVA) followed by Dunnett's multiple comparison test using total hydrolysate (TH) as control sample. Statistical analysis was performed using GraphPad Prism software (version 5.04).

## 3. Results and Discussion

### 3.2. Characteristics of the Enzymatic Hydrolysates and Their UF-Fractions

Table 1 reports the final DH values of EYP-TH and PPP-TH and their average solubility value over the pH range from 2 to 10. EYP-TH, obtained by the combined action of Alcalase and Protease N, is extensively hydrolyzed with a DH value of 25.3%. Alcalase is a non-specific bacterial endoprotease [36] whereas Protease N is a mixture of both proteases and peptidases. The combination of these enzymes thus led to an extensive hydrolysis EYP. The low DH value (5.2%) obtained for PPP-TH can be explained by the high specificity of trypsin, which cleaves at the carboxyl terminal of basic amino acids, Lys and Arg, and thus generates a limited number of peptides. It is also well-known that phosvitin's phosphoserine residues are arranged in a core section (residues 56-114), which forms blocks that can carry up to 15 consecutive residues alternated with the basic amino acids [37]. In such arrangements, the trypsin cleavage sites are presumably less accessible.

**Table 1:** Degree of hydrolysis and solubility of egg yolk proteins (EYP) and phosphoproteins (PPP) hydrolysates.

**Hydrolysate**	**Enzyme**	**Final DH (%)**	**Solubility (%)^a^**
EYP-TH	Alcalase + Protease N	25.3	> 98
PPP-TH	Trypsin	5.2	> 90

a Average solubility value measured from pH 2 until pH 10.

Table 1 also shows that solubility of both hydrolysates (EYP-TH and PPP-TH) was higher than 90% over the pH range from 2 to 10. Our observations are contrasting with those of Wang and Wang [20] who reported lower solubility values (50–60%) for EYP hydrolyzed with Protex 7L (from *Bacillus amyloliquefaciens*) and Protamax 1.5 (from *Bacillus licheniformis*), two bacterial proteases with low specificity. However, the high solubility values obtained for our hydrolysates can also be explained by the UF-removing of non-hydrolysed proteins and enzymes at the end of the enzymatic reaction [38].

Table 2 summarizes some compositional characteristics of EYP, PPP, their hydrolysates and UF-fractions. The compositional data for raw EYP and PPP were taken from our previous study [25]. The lower protein content of PPP-TH (34.7%) compared to EYP-TH (78.0%) can be explained by the lower DH value obtained upon tryptic hydrolysis of PPP, which yielded a TH containing larger peptides that would not permeate through the 10 kDa UF-membrane. In addition, the use of pH-stat technique during tryptic hydrolysis involved the addition of NaOH, which probably resulted in higher salt content in PPP-TH. The high phosphorus content of PPP (3.13%) compared to EYP (0.88) confirmed the effectiveness of the process used to concentrate the phosphoproteins from the delipidated EYP concentrate. However, the important decrease in phosphorus content of both hydrolysates suggests that a large proportion of phosphopeptides produced during the enzymatic hydrolysis cannot permeate through the 10 kDa UF-membrane used to stop the reaction. This decrease is higher for PPP-TH than for EYP-TH, and this observation can also be related to the lower DH value of PPP-TH and its content in larger peptides.

**Table 2:** Protein and phosphorus (P) contents (%, w/w dry basis) and N/P atomic ratio of egg yolk proteins, phosphoproteins, their enzymatic hydrolysates and UF-fractions.

	**Egg yolk proteins (EYP)**						**Phosphoproteins (PPP)**					
	
	**EYP**	**TH**	**UF5-R**	**UF5-P**	**UF1-R**	**UF1-P**	**PPP**	**TH**	**UF5-R**	**UF5-P**	**UF1-R**	**UF1-P**
Protein	77.3	78.0	75.9	71.3	71.2	72.8	61.9	34.7	43.9	23.7	37.0	19.0
P	0.88	0.40	0.33	0.32	0.42	0.32	3.13	1.04	0.89	1.23	1.26	1.23
N/P	31.1	69.1	81.4	78.9	60.1	80.6	7.0	11.8	17.5	6.8	10.4	5.5

UF-fractionation of EYP-TH led to little variations in protein (71.2–75.9%) and phosphorus (0.32–0.42%) contents of retentates and permeates fractions. The impact of UF-fractionation on protein and phosphorus contents was more pronounced for PPP-TH, but this can also be related to a more extended range of peptide molecular weights in this partial hydrolysate. Finally, the increased purity of phophopeptides in TH and UF-fractions was reflected by the decrease of N/P atomic ratio. For EYP, the lowest value was observed in UF1-R (60.1%) whereas for PPP, phophopeptides seemed to be concentrated in permeate fractions UF5-P (6.8%) and UF1-P (5.5%). These results thus suggested that phosphopeptides released during enzymatic hydrolysis of EYP and PPP varied in terms of molecular weight and seems to be shorter in PPP-TH.

**Figure 2:**
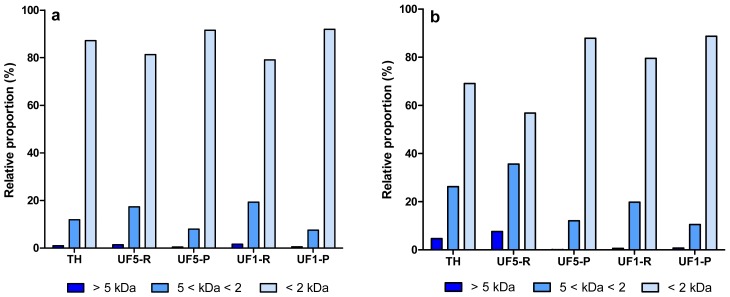
Molecular weight distribution profile (%) of protein/peptide components in total hydrolysates (TH) and their UF-fractions prepared from egg yolk proteins hydrolyzed with Alcalase and Protease N **(a)** and phophoproteins hydrolyzed with trypsin **(b)**.

The molecular weight (MW) distribution profile of protein/peptide components in enzymatic hydrolysates (EYP-TH and PPP-TH) and their UF-fractions are presented in Figure 2. Some differences between the MW distribution profiles of both hydrolysates were observed. EYP-TH and PPP-TH contained ∼85% and ∼70% of small peptides (<2 kDa), respectively. This observation is consistent with the higher DH value of EYP-TH (25.3%), which was hydrolysed with the combined action of Alcalase and Protease N (Table 1). Protein hydrolysis with Alcalase is known to yield a large proportion of small peptides with MW < 2 kDa [39]. Conversely, the content in large (>5 kDa) and intermediate MW peptides (5 < kDa < 2) were higher in PPP-TH, a partial tryptic hydrolysate of PPP. Goulas *et al.* [40] reported that tryptic digestion of PPP, such as phosvitin, resulted in a large fragment (Gln 49-Arg 212) and a small one (Ala 1-Arg 35). Similar observations were reported by Jiang and Mine [24].

The first UF-fractionation using the 5 kDa membrane removed a high proportion of large and intermediate MW peptides from PPP-TH whereas the other UF-fractions (UF5-P, UF1-R and UF1-P) obtained from both hydrolysates were relatively similar in terms of MW distribution profile of peptides.

The amino acid compositions of EYP, PPP, their TH and UF-fractions are reported in Table 3. The amino acid content was expressed as percentage of the total amino acids content, which was calculated on protein basis in order to take into account the differences in protein content between each extracts (Table 2).

As observed for phosphorus (Table 2), Ser content, presumably related with phophopeptides, was much higher in PPP (20.3%) than EYP (8.7%). However, Ser content decreased to relatively similar content in both hydrolysates (7–8%), which can be explained by the retention of phosphopeptides by the 10 kDa UF-membrane used to stop the enzymatic reaction. PPP also contained higher amount of His (4.0%) and Lys (9.1%) compared to EYP (His = 2.7%; Lys = 7.6%), and this difference was also reflected in their TH and UF-fractions but mainly for His content. Conversely, EYP was richer in Met, aromatic (Tyr, Phe) and hydrophobic amino acids (Val, Ile, Leu, Ala, Pro). However, the higher content of EYP in these amino acids could result from the much higher amount of Ser in PPP and from the fact that data are expressed as percentage of the total amino acids content. In fact, if the content in these amino acids between both hydrolysates are compared, only Ile and Leu contents remained higher in EYP-TH. Finally, even if EYP and PPP contained similar amount of Arg (5–6%), this amino acid seems to permeate through the 10 kDa UF-membrane for EYP-TH (6.4%), but not for PPP-TH (3.2%). In addition, the permeation of Arg through the 5 and 1 kDa UF-membranes showed an opposite behaviour for EYP and PPP.

**Table 3:** Amino acid composition (% of total amino acids, protein basis) of egg yolk proteins (EYP) and phosphoproteins (PPP), their hydrolysates and UF-fractions.

	**Egg yolk proteins (EYP)**						**Phosphoproteins (PPP)**					
	
	**EYP**	**TH**	**UF5-R**	**UF5-P**	**UF1-R**	**UF1-P**	**PPP**	**TH**	**UF5-R**	**UF5-P**	**UF1-R**	**UF1-P**
Thr	5.5	5.2	5.2	5.1	5.2	5.4	4.9	5.6	6.2	4.8	5.5	4.3
His	2.7	2.2	2.1	2.2	2.2	2.4	4.0	4.4	4.6	4.3	4.8	4.3
Lys	7.6	7.7	7.5	7.4	7.2	8.1	9.1	8.0	8.3	8.7	7.6	9.3
Tyr	4.9	4.6	4.4	4.8	4.6	5.4	2.9	4.8	4.6	4.3	4.8	4.3
Met	3.0	2.7	2.6	2.9	2.8	3.2	1.6	2.8	2.5	2.9	2.8	3.1
Val	6.3	6.3	6.2	6.5	6.2	7.0	4.4	6.8	6.8	6.3	6.5	6.2
Ile	5.4	5.2	5.0	5.5	5.2	5.4	2.9	4.8	1.5	4.4	4.8	4.9
Leu	9.6	9.7	8.8	10.3	9.6	11.3	5.1	8.4	8.3	8.7	8.3	9.3
Phe	4.9	4.6	4.4	4.8	4.6	5.4	3.1	4.8	4.6	5.3	5.2	5.6
Ser	8.7	6.9	7.1	6.7	6.5	7.0	20.3	8.0	8.3	6.7	7.2	6.8
Glu^1^	13.3	14.2	14.3	13.9	15.4	13.7	12.2	13.5	15.0	13.0	15.5	11.7
Ala	5.7	6.0	5.6	6.4	6.1	6.7	4.9	6.0	6.5	5.8	5.5	6.2
Pro	4.3	4.2	4.7	3.8	4.1	4.0	3.3	4.0	4.3	3.4	4.1	3.1
Arg	5.4	6.4	6.7	6.5	5.9	3.5	6.0	3.2	2.5	6.7	3.4	7.4
Gly	2.3	3.4	3.3	2.9	3.3	1.8	3.1	2.4	2.2	3.9	3.1	3.7
Asp^2^	10.3	10.8	12.0	10.1	11.1	9.4	12.4	12.7	13.8	11.1	11.0	9.9

Note: Values reported for Glu comprise Glu+Gln; Values reported for Asp comprise ASP+Asn.

### 3.2. Antioxidant Activity of Enzymatic Hydrolysates and Their UF-Fractions

**Figure 3:**
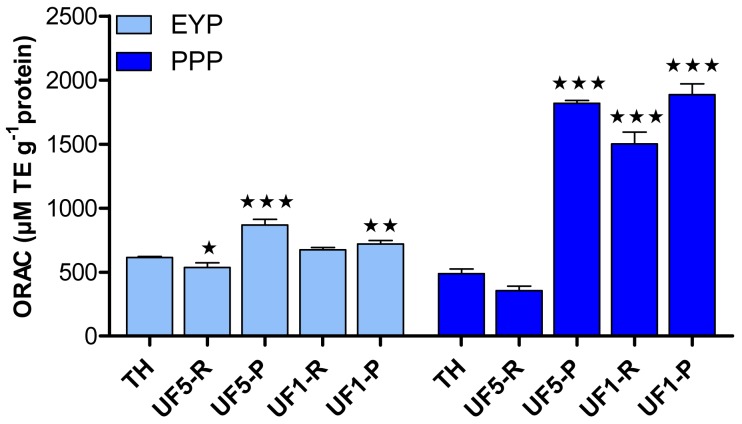
Antioxidant activity (μM TE·g^−1^ protein) of hydrolysates (TH) and UF-fractions prepared from egg yolk proteins (EYP) and phosphoproteins (PPP). Bars represent mean ± SD (*n* = 3) and statistical differences between TH and their respective UF-fractions are indicated (★ *p* < 0.05; ★★ *p* < 0.01; ★★★ *p* < 0.001).

Figure 3 shows the antioxidant activity of EYP and PPP hydrolysates and their UF-fractions as determined by the ORAC assay. In the present study, the ORAC data for delipidated EYP was not measurable due to its low solubility [20]. UF-fractionation of EYP-TH led to a significant increase of its antioxidant activity but only in permeate fractions. The highest increase in antioxidant capacity (*p* < 0.001) was observed for the permeate fraction obtained with the 5 kDa-membrane, for which ORAC value was 867.8 μM TE·g^−1^ protein compared to 613.1 μM TE·g^−1^ protein for the EYP-TH. The impact of UF-fractionation of PPP-TH on its antioxidant capacity was much more important, with ORAC values (1501.1–1886.8 μM TE·g^−1^ protein) more than 3 folds higher for all the UF-fractions comprising peptides with MW < 5 kDa compared to PPP-TH (489.2 μM TE·g^−1^ protein).

These results thus suggest that phosphoproteins contained in egg yolk could be responsible for its antioxidant activity, which also seems to be related to the molecular weight of peptides contained in the fractions. This is in agreement with the findings of Jiang and Mine [24] who established that antioxidant peptides obtained from hen egg phosvitin were characterized by a molecular weight value in the range of 1–3 kDa. It has also been shown [41,42] that the antioxidant activity of a protein hydrolysate increases with the degree of hydrolysis and with a higher proportion of smaller peptides. Similarly, egg yolk peptides containing two to three amino acids [17] and the lecithin-free egg yolk hydrolysates with molecular weight lower than 5 kDa [16] were the most efficient in inhibiting lipid oxidation. Two peptides of 10 and 15 amino acids were identified and both had Leu at their N-terminal positions.

**Figure 4:**
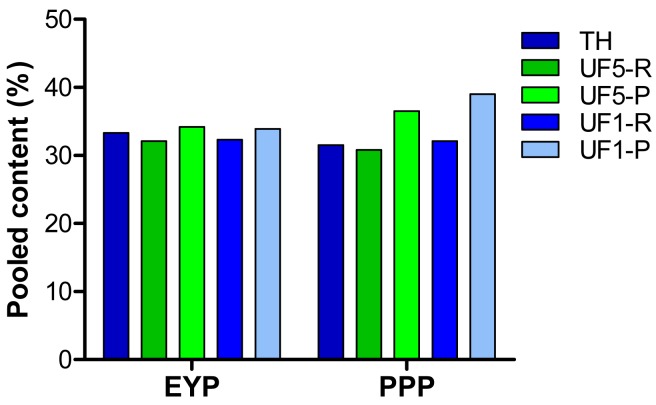
Pooled content (% of total amino acids, protein basis) in His, Lys, Met, Leu, Phe, and Arg of egg yolk proteins (EYP), phosphoproteins (PPP), their hydrolysates and UF-fractions.

In an attempt to establish a relationship between antioxidant activity of the different extracts (Figure 3) and their amino acid composition (Table 3), calculations of the total content in specific (basic, acid, polar, non-polar, hydroxylated, aromatic) or known antioxidant amino acids (Met, Tyr, Phe, His, Leu, Pro) were made. These calculations revealed that amino acids for which the total most reflected the antioxidant activity measured for the different extracts were His, Lys, Met, Leu, Phe and Arg as illustrated in Figure 4.

The higher antioxidant capacity measured UF-permeates fractions (1 kDa and 5 kDa) compared to retentates (Figure 3) may result from the higher proportion of pooled concentrations in His, Met, Leu, Phe, Arg and Lys in permeates (Figure 4). Among these amino acids, His, Met, Leu and Phe have been recognized as antioxidant [5]. However, the concentrations in Lys and Arg were higher in the permeates than in the retentates as a result of the specificity of trypsin, but this may not be directly influencing the antioxidant activity of the UF-fractions.

Overall, our data on amino acid composition and phosphorus content of the UF-fractions provide some evidence about the contribution of specific amino acids and phosphorus content to the antioxidant activity of PPP's. In addition, our results suggest that PPP UF-fractions containing peptides with molecular weight lower than 5 kDa (UF5-P, UF1-R and UF1-P) and having the highest phosphorus content may contain the bioactive peptides. However, the relative contribution of molecular weight, phosphorus content and amino acid profile to the antioxidant activity cannot be established from the present study.

## 4. Conclusions

The *in vitro* antioxidant activity of the UF-permeates was significantly higher than those of the non-fractionated hydrolysates. The findings from the present study suggest that tryptic hydrolysis of egg phosvitin combined to UF-fractionation of hydrolysate could provide new opportunities for the development of health-promoting ingredients.
